# *catena*-Poly[[(6-carb­oxy­pyrazine-2-carboxyl­ato)lithium]-μ-aqua]

**DOI:** 10.1107/S1600536811046198

**Published:** 2011-11-09

**Authors:** Wojciech Starosta, Janusz Leciejewicz

**Affiliations:** a Institute of Nuclear Chemistry and Technology, ul. Dorodna 16, 03-195 Warszawa, Poland

## Abstract

The asymmetric unit of the title compound, [Li(C_6_H_3_N_2_O_4_)(H_2_O)]_*n*_, contains an Li^I^ ion with a distorted trigonal–bipyramidal coordination environment. It is chelated by a singly protonated ligand mol­ecule *via* its heterocyclic N atom, by two O aoms, each donated by an adjacent carboxyl­ate group, and is further coordinated by a water O atom which acts as a bridge, forming a mol­ecular ribbon. A proton attached to one of the carboxyl­ate O atoms is situated on an inversion centre and forms a short centrosymmetric hydrogen bond, generating mol­ecular layers parallel to the *ac* plane. These layers are held together by weak O—H⋯O hydrogen bonds in which the coordinated water mol­ecules act as donors, whereas carboxyl­ate O atoms are acceptors.

## Related literature

For the structures of three lithium complexes with pyrazine-2,3-dicarboxyl­ate and water ligands, see: Tombul *et al.* (2008 ▶); Tombul & Guven (2009) ▶; Starosta & Leciejewicz (2011*b* ▶). For the structure of a Li^I^ complex with a pyrazine-2,5-dicarboxyl­ate ligand, see: Starosta & Leciejewicz (2011*a* ▶) and for the structure of a Li^I^ complex with pyrazine-2,3,5,6-tetra­carboxyl­ate, see: Starosta & Leciejewicz (2010 ▶). The structure of pyrazine-2,6-dicarboxyl­ate acid dihydrate has been also reported, see: Ptasiewicz-Bąk & Leciejewicz (2003 ▶). 
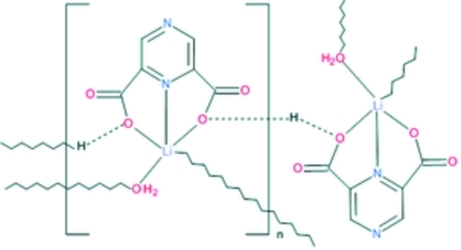


## Experimental

### 

#### Crystal data

[Li(C_6_H_3_N_2_O_4_)(H_2_O)]
*M* *_r_* = 192.06
Monoclinic, 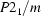

*a* = 3.5346 (7) Å
*b* = 12.519 (3) Å
*c* = 8.3583 (17) Å
β = 97.86 (3)°
*V* = 366.37 (13) Å^3^
*Z* = 2
Mo *K*α radiation
μ = 0.15 mm^−1^
*T* = 293 K
0.31 × 0.22 × 0.08 mm

#### Data collection

Kuma KM-4 four-circle diffractometer
Absorption correction: analytical (*CrysAlis RED*; Oxford Diffraction, 2008 ▶) *T*_min_ = 0.954, *T*_max_ = 0.973
1262 measured reflections
1106 independent reflections
729 reflections with *I* > 2σ(*I*)
*R*_int_ = 0.027
3 standard reflections every 200 reflections intensity decay: 1.3%

#### Refinement

*R*[*F* ^2^ > 2σ(*F* ^2^)] = 0.054
*wR*(*F* ^2^) = 0.171
*S* = 1.09
1106 reflections
75 parameters
2 restraints
H atoms treated by a mixture of independent and constrained refinement
Δρ_max_ = 0.38 e Å^−3^
Δρ_min_ = −0.31 e Å^−3^

### 

Data collection: *KM-4 Software* (Kuma, 1996 ▶); cell refinement: *KM-4 Software*; data reduction: *DATAPROC* (Kuma, 2001 ▶); program(s) used to solve structure: *SHELXS97* (Sheldrick, 2008 ▶); program(s) used to refine structure: *SHELXL97* (Sheldrick, 2008 ▶); molecular graphics: *SHELXTL* (Sheldrick, 2008 ▶); software used to prepare material for publication: *SHELXTL*.

## Supplementary Material

Crystal structure: contains datablock(s) I, global. DOI: 10.1107/S1600536811046198/kp2364sup1.cif

Structure factors: contains datablock(s) I. DOI: 10.1107/S1600536811046198/kp2364Isup2.hkl

Additional supplementary materials:  crystallographic information; 3D view; checkCIF report

## Figures and Tables

**Table 1:** Selected bond lengths (Å)

N1—Li1	2.115 (7)
O1—Li1	2.271 (2)
O3—Li1	1.950 (7)
O3—Li1^i^	2.085 (7)
Li1—O1^ii^	2.271 (2)

Symmetry codes: (i) 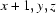; (ii) 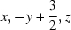.

**Table 2:** Hydrogen-bond geometry (Å, °)

*D*—H⋯*A*	*D*—H	H⋯*A*	*D*⋯*A*	*D*—H⋯*A*
O3—H31⋯O2^iii^	0.83 (2)	2.24 (2)	2.9987 (19)	152 (3)
O1—H1⋯O1^iii^	1.23 (1)	1.23 (1)	2.455 (3)	180 (1)

Symmetry code: (iii) 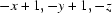.

**Fig. 1.**
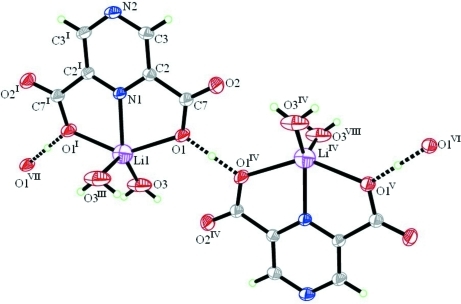
The asymmetric unit of the title compound with atom labelling scheme and 50% probability displacement ellipsoids. Symmetry code: ix, -y + 3/2, z; iix + 1, y, z; iiix - 1, y, z; iv 1 - x, 1 - y, -z; v 1 - x, -1/2 + y, -z; vix, 1/2 - y, z; vii 1 - x, 1/2 + y, -z; viii 2 - x, 1 - y, -z.

**Fig. 2.**
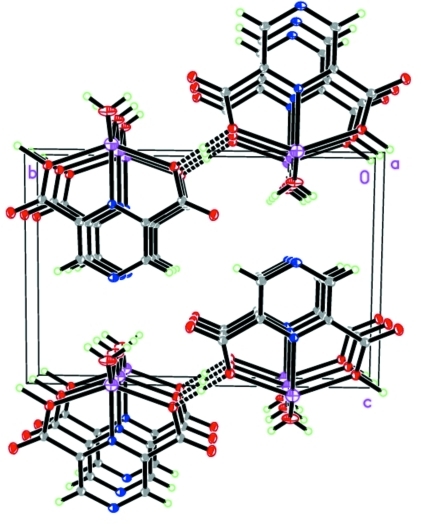
The alignment of the ribbons viewed along the axis a.

## supplementary crystallographic information

### Comment

The asymmetric unit of the title compound consists of a Li^I^ ion, a singly deprotonated pyrazine-2,6-dicarboxylate iigand molecule and a coordinated water molecule (Fig. 1). The coordination environment of the Li1 ion is composed of five atoms: ligand carboxylate O1, O1^i^, hetero-ring N1, aqua O3 and O3^iii^ atoms. The coplanar Li1, N1, O3 and O3^iii^ form the base of a distorted trigonal bipyramid with O1 and O1^i^ atoms at its apices.[Symmetry code: ^i^*x*, -*y* + 3/2, *z*; ^ii^*x* + 1, *y*, *z*, ^iii^*x* - 1, *y*, *z*, ^iv^ 1 - *x*, 1 - *y*, -*z*]. The observed Li—O and Li—N bond distances (Table 1) are typical for Li^I^ complexes with diazine carboxylate ligands, see, for example: Tombul & Guven, (2009); Starosta & Leciejewicz, (2010); Starosta & Leciejewicz,(2011*b*). Coordinated aqua O3 atom bridges Li1 with Li^ii^ ion to form molecular ribbons which propagate in the crystal alon [001] direction (Fig. 2). The carboxylato O1 atom remains protonated and mantains the charge balance. This proton, located at an inversion centre, forms a short centrosymmetric O1—H1···O1^iv^ hydrogen bond of 2.455 (3) A° which links adjacent ribbons to form molecular layers. The pyrazine ring is planar with r.m.s of 0.0024 (1) Å.The C7/O1/O2 and C7^i^/O1^i^/O2^i^ carboxylic groups make with it dihedral angles of 3.0 (1)°. Bond distances and bond angles within the ligand molecule do not differ from those reported in the structure of pyrazine-2,6-dicarboxylic acid dihydrate (Ptasiewicz-Bąk & Leciejewicz, 2003). The layers are held together by weak hydrogen bonds in which the coordinated water molecules act as donors and carboxylate O atoms and hetero-ring N atoms from adjacent layers are as acceptors (Table 2). Protonated ligand carboxylate groups have been observed in the structures of Li^I^ complexes with pyrazine-2,3-carboxylate (Tombul *et al.*, 2008, Starosta & Leciejewicz, 2011*b*) and pyrazine-2,5-dicarboxylate (Starosta & Leciejewicz, 2011*a*) ligands and in the structure of a Li^I^ complex with pyrazine-2,3,5,6-tetracarboxylate ligand (Starosta & Leciejewicz, 2010). In the above structures, protons participate in short hydrogen bonds in which O atoms from adjacent intra-ligand carboxylate groups are donors and acceptors.

### Experimental

Hot aqueous solutions of 1 mmol of pyrazine-2,6-dicarboxylic acid dihydrate and 1 mmol of lithium hydroxide (Aldrich) were mixed and boiled under reflux with constant stirring for 6 h. Left for evaporation at room temperature, after a couple of days small single-crystal plates of the title complex were obtained. Crystals were washed with cold ethanol and dried in air.

### Refinement

Pyrazine ring H atoms atoms were placed in calculated positions with C—H = 0.93 and 0.96Å and treated as riding on the parent atoms with *U*_iso_(H)= 1.2*U*_eq_(C)or *U*_iso_(H)=1.5*U*_eq_(C_methyl_). Water H atoms were found in Fourier map and refined isotropically.

### Crystal data

**Table:**

[Li(C_6_H_3_N_2_O_4_)(H_2_O)]	*F*(000) = 196
*M**_r_* = 192.06	*D*_x_ = 1.741 Mg m^−^^3^
Monoclinic, *P*2_1_/*m*	Mo *K*α radiation, λ = 0.71073 Å
Hall symbol: -P 2yb	Cell parameters from 25 reflections
*a* = 3.5346 (7) Å	θ = 6–15°
*b* = 12.519 (3) Å	µ = 0.15 mm^−^^1^
*c* = 8.3583 (17) Å	*T* = 293 K
β = 97.86 (3)°	Plates, colourless
*V* = 366.37 (13) Å^3^	0.31 × 0.22 × 0.08 mm
*Z* = 2	

### Data collection

**Table:**

Kuma KM-4 four-circle diffractometer	729 reflections with *I* > 2σ(*I*)
Radiation source: fine-focus sealed tube	*R*_int_ = 0.027
graphite	θ_max_ = 30.1°, θ_min_ = 3.0°
Profile data from ω/2θ scans	*h* = 0→4
Absorption correction: analytical (*CrysAlis RED*; Oxford Diffraction, 2008)	*k* = −17→0
*T*_min_ = 0.954, *T*_max_ = 0.973	*l* = −11→11
1262 measured reflections	3 standard reflections every 200 reflections
1106 independent reflections	intensity decay: 1.3%

### Refinement

**Table:**

Refinement on *F*^2^	Primary atom site location: structure-invariant direct methods
Least-squares matrix: full	Secondary atom site location: difference Fourier map
*R*[*F*^2^ > 2σ(*F*^2^)] = 0.054	Hydrogen site location: inferred from neighbouring sites
*wR*(*F*^2^) = 0.171	H atoms treated by a mixture of independent and constrained refinement
*S* = 1.09	*w* = 1/[σ^2^(*F*_o_^2^) + (0.1039*P*)^2^ + 0.0995*P*] where *P* = (*F*_o_^2^ + 2*F*_c_^2^)/3
1106 reflections	(Δ/σ)_max_ < 0.001
75 parameters	Δρ_max_ = 0.38 e Å^−^^3^
2 restraints	Δρ_min_ = −0.31 e Å^−^^3^

### Special details

**Table:**

Geometry. All e.s.d.'s (except the e.s.d. in the dihedral angle between two l.s. planes) are estimated using the full covariance matrix. The cell e.s.d.'s are taken into account individually in the estimation of e.s.d.'s in distances, angles and torsion angles; correlations between e.s.d.'s in cell parameters are only used when they are defined by crystal symmetry. An approximate (isotropic) treatment of cell e.s.d.'s is used for estimating e.s.d.'s involving l.s. planes.
Refinement. Refinement of *F*^2^ against ALL reflections. The weighted *R*-factor *wR* and goodness of fit *S* are based on *F*^2^, conventional *R*-factors *R* are based on *F*, with *F* set to zero for negative *F*^2^. The threshold expression of *F*^2^ > σ(*F*^2^) is used only for calculating *R*-factors(gt) *etc*. and is not relevant to the choice of reflections for refinement. *R*-factors based on *F*^2^ are statistically about twice as large as those based on *F*, and *R*- factors based on ALL data will be even larger.

### Fractional atomic coordinates and isotropic or equivalent isotropic displacement parameters (Å^2^)

**Table:**

	*x*	*y*	*z*	*U*_iso_*/*U*_eq_	
N1	0.2901 (6)	0.7500	0.2305 (2)	0.0216 (4)	
O1	0.4179 (5)	0.57853 (10)	0.07619 (15)	0.0333 (4)	
C2	0.2425 (5)	0.65866 (13)	0.30619 (19)	0.0216 (4)	
N2	0.0883 (7)	0.7500	0.5385 (2)	0.0297 (5)	
O2	0.2587 (5)	0.47052 (12)	0.27081 (17)	0.0371 (4)	
C3	0.1409 (5)	0.65888 (14)	0.4618 (2)	0.0269 (4)	
H3	0.1092	0.5942	0.5130	0.032*	
C7	0.3068 (5)	0.55822 (14)	0.2144 (2)	0.0245 (4)	
O3	0.8304 (9)	0.7500	−0.1306 (3)	0.0572 (8)	
Li1	0.3902 (17)	0.7500	−0.0132 (8)	0.0456 (13)	
H31	0.866 (12)	0.6976 (8)	−0.186 (4)	0.092 (14)*	
H1	0.5000	0.5000	0.0000	0.10 (2)*	

### Atomic displacement parameters (Å^2^)

**Table:**

	*U*^11^	*U*^22^	*U*^33^	*U*^12^	*U*^13^	*U*^23^
N1	0.0281 (10)	0.0194 (9)	0.0187 (8)	0.000	0.0084 (7)	0.000
O1	0.0561 (9)	0.0228 (7)	0.0254 (6)	0.0003 (6)	0.0216 (6)	−0.0014 (5)
C2	0.0253 (8)	0.0206 (7)	0.0198 (7)	−0.0006 (6)	0.0059 (5)	0.0012 (6)
N2	0.0404 (12)	0.0314 (12)	0.0196 (9)	0.000	0.0124 (8)	0.000
O2	0.0584 (10)	0.0223 (7)	0.0340 (7)	0.0004 (6)	0.0186 (6)	0.0037 (5)
C3	0.0348 (9)	0.0261 (9)	0.0217 (7)	0.0000 (7)	0.0109 (6)	0.0031 (6)
C7	0.0300 (8)	0.0223 (7)	0.0225 (7)	0.0009 (6)	0.0080 (6)	0.0002 (6)
O3	0.0642 (18)	0.084 (2)	0.0247 (10)	0.000	0.0122 (10)	0.000
Li1	0.039 (3)	0.053 (3)	0.046 (3)	0.000	0.007 (2)	0.000

### Geometric parameters (Å, °)

**Table:**

N1—C2^i^	1.3287 (18)	O2—C7	1.216 (2)
N1—C2	1.3287 (18)	C3—H3	0.9300
N1—Li1	2.115 (7)	O3—Li1	1.950 (7)
O1—C7	1.295 (2)	O3—Li1^ii^	2.085 (7)
O1—Li1	2.271 (2)	O3—H31	0.825 (17)
O1—H1	1.2275 (13)	Li1—O3^iii^	2.085 (7)
C2—C3	1.396 (2)	Li1—O1^i^	2.271 (2)
C2—C7	1.506 (2)	Li1—Li1^iii^	3.5346 (7)
N2—C3^i^	1.334 (2)	Li1—Li1^ii^	3.5346 (7)
N2—C3	1.334 (2)		
			
C2^i^—N1—C2	118.8 (2)	O3—Li1—N1	137.3 (3)
C2^i^—N1—Li1	120.51 (10)	O3^iii^—Li1—N1	100.4 (3)
C2—N1—Li1	120.51 (10)	O3—Li1—O1^i^	99.45 (16)
C7—O1—Li1	118.33 (19)	O3^iii^—Li1—O1^i^	98.65 (16)
C7—O1—H1	115.31 (13)	N1—Li1—O1^i^	71.83 (16)
Li1—O1—H1	126.08 (17)	O3—Li1—O1	99.45 (16)
N1—C2—C3	120.51 (16)	O3^iii^—Li1—O1	98.65 (16)
N1—C2—C7	115.98 (14)	N1—Li1—O1	71.84 (16)
C3—C2—C7	123.52 (15)	O1^i^—Li1—O1	141.9 (3)
C3^i^—N2—C3	117.5 (2)	O3—Li1—Li1^iii^	150.10 (19)
N2—C3—C2	121.34 (16)	O3^iii^—Li1—Li1^iii^	27.79 (19)
N2—C3—H3	119.3	N1—Li1—Li1^iii^	72.60 (17)
C2—C3—H3	119.3	O1^i^—Li1—Li1^iii^	89.89 (15)
O2—C7—O1	126.77 (16)	O1—Li1—Li1^iii^	89.89 (15)
O2—C7—C2	121.16 (15)	O3—Li1—Li1^ii^	29.90 (19)
O1—C7—C2	112.07 (15)	O3^iii^—Li1—Li1^ii^	152.21 (18)
Li1—O3—Li1^ii^	122.3 (3)	N1—Li1—Li1^ii^	107.40 (17)
Li1—O3—H31	119 (3)	O1^i^—Li1—Li1^ii^	90.11 (15)
Li1^ii^—O3—H31	93 (3)	O1—Li1—Li1^ii^	90.11 (15)
O3—Li1—O3^iii^	122.3 (3)	Li1^iii^—Li1—Li1^ii^	179.999 (1)

Symmetry codes: (i) *x*, −*y*+3/2, *z*; (ii) *x*+1, *y*, *z*; (iii) *x*−1, *y*, *z*.

### Hydrogen-bond geometry (Å, °)

**Table:**

*D*—H···*A*	*D*—H	H···*A*	*D*···*A*	*D*—H···*A*
O3—H31···O2^iv^	0.83 (2)	2.24 (2)	2.9987 (19)	152 (3)
O1—H1···O1^iv^	1.23 (1)	1.23 (1)	2.455 (3)	180.(1)

Symmetry codes: (iv) −*x*+1, −*y*+1, −*z*.
